# Pharmacokinetics and pharmacodynamics of anti-tuberculosis drugs: An evaluation of *in vitro*, *in vivo* methodologies and human studies

**DOI:** 10.3389/fphar.2022.1063453

**Published:** 2022-12-09

**Authors:** Jan-Willem C. Alffenaar, Jurriaan E. M. de Steenwinkel, Andreas H. Diacon, Ulrika S. H. Simonsson, Shashikant Srivastava, Sebastian G. Wicha

**Affiliations:** ^1^ Sydney Institute for Infectious Diseases, The University of Sydney, Sydney, NSW, Australia; ^2^ School of Pharmacy, The University of Sydney Faculty of Medicine and Health, Sydney, NSW, Australia; ^3^ Westmead Hospital, Sydney, NSW, Australia; ^4^ Department of Medical Microbiology and Infectious Diseases, Erasmus MC, Rotterdam, Netherlands; ^5^ TASK, Cape Town, South Africa; ^6^ Department of Pharmaceutical Biosciences, Uppsala University, Uppsala, Sweden; ^7^ Department of Pulmonary Immunology, University of Texas Health Science Center at Tyler, Tyler, TX, United States; ^8^ Department of Clinical Pharmacy, Institute of Pharmacy, University of Hamburg, Hamburg, Germany

**Keywords:** tuberculosis, pharmacokinetics, pharmacodynamics, modeling and simulation, dose optimization, anti-TB drugs

## Abstract

There has been an increased interest in pharmacokinetics and pharmacodynamics (PKPD) of anti-tuberculosis drugs. A better understanding of the relationship between drug exposure, antimicrobial kill and acquired drug resistance is essential not only to optimize current treatment regimens but also to design appropriately dosed regimens with new anti-tuberculosis drugs. Although the interest in PKPD has resulted in an increased number of studies, the actual bench-to-bedside translation is somewhat limited. One of the reasons could be differences in methodologies and outcome assessments that makes it difficult to compare the studies. In this paper we summarize most relevant *in vitro*, *in vivo*, *in silico* and human PKPD studies performed to optimize the drug dose and regimens for treatment of tuberculosis. The *in vitro* assessment focuses on MIC determination, static time-kill kinetics, and dynamic hollow fibre infection models to investigate acquisition of resistance and killing of Mycobacterium tuberculosis populations in various metabolic states. The *in vivo* assessment focuses on the various animal models, routes of infection, PK at the site of infection, PD read-outs, biomarkers and differences in treatment outcome evaluation (relapse and death). For human PKPD we focus on early bactericidal activity studies and inclusion of PK and therapeutic drug monitoring in clinical trials. Modelling and simulation approaches that are used to evaluate and link the different data types will be discussed. We also describe the concept of different studies, study design, importance of uniform reporting including microbiological and clinical outcome assessments, and modelling approaches. We aim to encourage researchers to consider methods of assessing and reporting PKPD of anti-tuberculosis drugs when designing studies. This will improve appropriate comparison between studies and accelerate the progress in the field.

## 1 Introduction

After diagnosis, providing each tuberculosis (TB) patient with the right drugs at the right dose for the right duration in the right combination is important to effectively reduce transmission, prevent relapse and control the risk of development of drug resistance (Alffenaar et al., 2022). Following other fields in infectious diseases there has been an increased interest in pharmacokinetics and pharmacodynamics (PKPD) of anti-tuberculosis drugs. A better understanding of the relationship between drug exposure and antimicrobial kill and acquired drug resistance is essential not only to optimize current standard of care regimen but also to design more appropriate dosing regimens for new and repurposed drugs with anti-tuberculosis activity (Peloquin and Davies, 2021). PKPD studies can help to determine which drug exposure indices is best associated with antimicrobial kill as well as suppression of resistance development during the course of therapy. As for any antimicrobial drugs the drug exposure indices associated with the response of *Mycobacterium tuberculosis* to the drug are the area under the unbound drug concentration-time curve over the minimal inhibitory concentration (*f*AUC/MIC), the maximum concentration over the MIC (C_max_/MIC) and the time the unbound drug concentration exceeds the MIC (%*f*T > MIC). There is a broad range in PKPD studies which are ideally complementary in providing relevant data to optimize the drug dose and regimens for treatment of TB.

*In vitro* studies are focused on MIC determination, static time-kill kinetics and/or dynamic models that allow to mimic a pharmacokinetic profile *in vitro* such as the hollow fibre infection model (Gumbo and Alffenaar, 2018). These models can include various metabolic populations and strains of *Mycobacterium tuberculosis* (Goossens et al., 2021). Besides assisting in drug development they are able to mimic specific real-life conditions to explore effects on treatment response and acquired drug resistance (Srivastava et al., 2011a). *In vivo* studies able to reflect the pathophysiological conditions of the TB infections study the effect of different drug dosages—exposure on treatment outcome including relapse and drug resistance. Human PKPD is characterized in phase 2a early bactericidal activity (EBA) studies and should be part of Phase 2b-3 studies to further refine the understanding of the exposure-response relationship (Martson et al., 2021). In addition to clinical trials, studies under operational research conditions are relevant to capture drug dosing, exposure, and treatment response under programmatic care (Alffenaar et al., 2020). Modelling and simulation approaches are very helpful to 1) evaluate the population PK of a TB drug in a population of interest, 2) characterize the exposure-response relationship of TB drugs, 3) elucidate exposure-safety profiles, and 4) can link the data from pre-clinical and clinical studies to better understand the drug exposure effect relationship (Märtson et al., 2020).

Although the interest in PKPD has resulted in an increased number of PKPD studies, the actual bench-to-bedside translation is somewhat limited. Firstly, current regimens have by necessity been developed using “trial and error” approaches. This resulted in practically relevant treatment regimens that have stood the test of time but did not create data that would assist in validating today’s new preclinical methods that must await further large trials in humans that will determine the value of the predictions made. Secondly, differences in methodologies and outcome assessments make it difficult to compare the studies and link preclinical finding to patient outcomes, which are further complicated by organizational gaps that preclude consequent utilization of pre-clinical data to optimally inform clinical trials. For instance, preclinical combination experiments are carried out with different combination partner drugs than those that are used in clinical trials. Thirdly, costs of phase 3 clinical trials as well as uptake of trials results in guidelines and subsequent implementation in practice challenge the stakeholders’ capacity.

To improve the synthesis of PKPD information from preclinical and clinical PKPD studies this review aims to describe the concept of different studies, optimized study design considering drug properties and study aims, importance of uniform reporting including microbiological and clinical outcome assessment, and possible modelling parameters. The information on uniformed methods of assessing and reporting PK/PD parameters of anti-tuberculosis drugs should accelerate the translation to clinical practice.

## 2 Materials and methods

A non-systematic review of English literature was performed in PubMed and Web of Science. Keywords included *in vivo, in vitro*, early bactericidal activity, pharmacokinetics, pharmacodynamics, tuberculosis, murine model, pharmacometrics, limited sampling. Information retrieved was summarized to describe the concept of different studies and recommendations were provided on optimized study design considering drug properties and study aims, importance of uniform reporting including microbiological and clinical outcome assessment, and possible modelling parameters.

## 3 *In vitro* pharmacokinetics/pharmacodynamics

Understanding the antimicrobial activity spectrum of the drug is the first key step in preclinical PKPD. Using wide concentration range MIC experiments are performed for which broth micro-dilution is most commonly used and recommended method (Wayne, 2018). By using a broad variety of clinical strains, gathered from around the world, a view on the MIC distribution is obtained. The next step involves the determination of the efficacy and potency of the extent of the drug by performing time-kill kinetic studies, either at static concentrations in the test-tubes or fluctuating concentrations using dynamic models. (de Steenwinkel et al., 2010) In the static time-kill (STK) studies the drug concentration remains fixed over time and the bacterial response are measured in terms change on the optical density (OD_A600_) and/or colony forming units (CFU). The STK studies are commonly performed using the actively replicating logarithmic phase bacteria in cultures and based on the extent of kill drugs are commonly classified as bactericidal or bacteriostatic (Wald-Dickler et al., 2018).

However, in a lung lesion, *M. tuberculosis* can be present under different metabolic states (Mitchison, 1979), such as actively replicating extracellular bacteria, intracellular bacteria inside the macrophage, slowly replicating bacteria under acidic condition in the caseum, or non-replicating persisters for which the exact location is hard to determine. Since in the STK studies the drug concentrations do not change over time as it is expected *in vivo*, dynamic models had to be developed to allow for drug concentrations to be actively changed over time. One such dynamic model is the hollow fibre model system of tuberculosis (HFS-TB) (Gumbo et al., 2004; Gumbo et al., 2005; Gumbo et al., 2007a; Gumbo et al., 2007b; Srivastava and Gumbo, 2012). The HFS-TB consist of a cartridge housing multiple hollow fiber capillary tubes with pore size such that bacteria cannot cross the membranes. This creates two compartments in the cartridge; an intra-capillary compartment through which drug and nutrients in the media flows across the membrane, and an extra-capillary compartment where the bacteria come into contact with drug containing media. The drug is infused into the central compartment of the HFS-TB *via* programable syringe pump, and the time-to-maximum concentration (T_max_) can be varied to mimic human-like PK as well as the dosing frequency. Further, the fresh media is continuously infused into the systems using peristaltic pumps and at the same time the media coming out of the cartridge is pumped out using a second set of peristaltic pumps. The dilution rate, using the peristaltic pumps, determines the half-life of the drug in the HFS-TB. Multiple half-lives of the drugs in a combination regimen can be achieved in the HFS-TB, by adjusting the parameters on the programable syringe pumps (Srivastava et al., 2011b; Deshpande et al., 2016). Another dynamic model was developed making use of a central compartment containing a liquid bacteria culture. In the model developed by Vaddady et al. (2010), new medium and/or drug in increasing concentration is added to the central compartment while removing the medium with the drugs is performed *via* a filter leaving the bacteria in place. In both models a sampling port is available to monitor bacterial count and drug concentration over 24 h to validate the concentration-time profile of the drugs in the central compartment, inside the cells (in case of studying intracellular metabolic population), as well as intrabacterial drug concentrations (Vaddady et al., 2010; Deshpande et al., 2019b). Thus, the dynamic models offer an advantage over the STK experiments in terms of precise control of drug concentration-time profiles to which *M. tuberculosis* is exposed as well as longer study duration, this permits to study development of drug resistance during the therapy. However, the dynamic models are limited by relatively low through-put and often unavailable human PK data to set the model parameters correctly.

Given that physical barriers to drug penetration to the anatomical sites (Dheda et al., 2018) and physiological conditions (e.g., pH; attributed to activity of pyrazinamide (McDERMOTT and TOMPSETT, 1954) and recently contradicted (Gumbo et al., 2009; Srivastava et al., 2016b; Lanoix et al., 2016) can affect the efficacy of the drug, the HFS-TB can also be used the PKPD relationship against different *M. tuberculosis* metabolic populations (Gumbo et al., 2009; Srivastava et al., 2011b; Srivastava et al., 2016b; Srivastava et al., 2021b; Ruth et al., 2022). Moreover, it is the non-protein bound (free) drug concentration that exerts the antimicrobial effect. Although highly protein bound drugs may have low unbound concentrations in plasma, accumulation of these drugs at the site of infection as well as intracellularly results in concentrations which are high enough to kill *M. tuberculosis.* (Srivastava et al., 2020) The HFS-TB offers a controlled environment where the hollow fiber membrane material can be changed as per the drugs physicochemical properties and the protein content in the media can be adjusted to minimize the drug binding. Thus, the concentration used in each HFS-TB experiments represent the free drug concentrations. This means that after appropriate adjustments even highly protein bound drugs like bedaquiline can be successfully studied in the HFS-TB.

Finally, to determine the optimal clinical dose to achieve the HFS-TB PK/PD parameter optimized drug exposure and determine the susceptibility breakpoint, *in silico* Monte-Carlo clinical trial simulations are used (D’Argenio and Schumitzky, 1997; D’Argenio et al., 2009). A systemic analysis of the published HFS-TB studies concluded that this dynamic model in combination with *in silico* simulations could predict the clinical outcomes of dose optimization and target attainment in combination regimens fairly accurately (Cavaleri and Manolis, 2015; Chilukuri et al., 2015) and these findings were also included in the recently published WHO technical report (World Health Organization, 2018) as well as clinical standard for tuberculosis (Alffenaar et al., 2022). In summary (Table 1), dynamic pre-clinical models are useful in the tuberculosis drug development space with the ability to inform future clinical trial design by providing PKPD based proof-of-concept for developing drugs as a single agent or in combination where each drug dose is selected to achieve the pharmacodynamic target (C_max_/MIC, AUC/MIC, %T > MIC) of the drugs in the regimen. In addition, data from static and dynamic hollow-fibre infection models can be used to develop model-based PKPD relationships linked to human population PK models to support dose selection for first-in-man studies and/or selection of optimized drug combinations. (Wicha et al., 2018)

**TABLE 1:** HFS-TB derived drug exposure target and susceptibility breakpoint MIC of drugs with antimycobacterial activity.

Drug	PK/PD target	Susceptibility breakpoint (mg/L)	References
Amikacin	C_max_/MIC >10.13		Srivastava et al. (2016a)
Cefazolin	%T > MIC = 46.76	1–2	Srivastava et al. (2021c)
Cefdinir	AUC_0-24_/MIC = 578.86		Srivastava et al. (2021d)
Ceftriaxone	%T > MIC = 68		Srivastava et al. (2020)
Cycloserine	%T > MIC = 30		Deshpande et al. (2018a)
Delamanid	AUC_0-24_/MIC = 195		Mallikaarjun et al. (2021)
Ertapenem	%T > MIC >40	2	van Rijn et al. (2017)
Ethambutol	C_max_/MIC >0.51	4	Srivastava et al. (2010)
Ethionamide	AUC_0-24_/MIC >56.2	2.5	Deshpande et al. (2018c)
Faropenem	%T > MIC >62		Deshpande et al. (2016)
Gatifloxacin	AUC_0-24_/MIC >184	0.5	Deshpande et al. (2018d)
Isoniazid	AUC_0-24_/MIC >567	0.0312	Gumbo et al. (2007c)
Levofloxacin	AUC_0-24_/MIC >146	0.5	Deshpande et al. (2018b)
Linezolid	AUC_0-24_/MIC >119	2	Srivastava et al. (2017)
Minocycline	AUC_0-24_/MIC = 71.58	8	Deshpande et al. (2019b)
Moxifloxacin	AUC_0-24_/MIC >56	1	Gumbo et al. (2004)
Penicillin	%T > MIC >65		Deshpande et al. (2018e)
Pyrazinamide	AUC_0-24_/MIC >209	50	Gumbo et al. (2009)
Rifampin	AUC_0-24_/MIC >1,360	0.0625	(Gumbo et al., 2007a; Gumbo, 2010)
Tedizolid	AUC_0-24_/MIC >200	0.5	Srivastava et al. (2018)
Thioridazine	AUC_0-24_/MIC = 50.53		Musuka et al. (2013)
Tigecycline	AUC_0-24_/MIC >42.3		Deshpande et al. (2019a)
Vancomycin	AUC_0-24_/MIC = 2,954		Srivastava et al. (2021a)

Drugs are arranged in alphabetical order. MIC, minimum inhibitory concentration; Cmax, peak concentration; AUC, area under the concentration-time curve; %T > MIC, percentage of time drug concentration persist above MIC.

## 4 *In vivo* pharmacokinetics/pharmacodynamics

*In vivo* studies can be performed in sequentially or simultaneously with *in vitro* studies. A reasonable strategy that considers animal welfare, minimizes complexity and saves cost is to move only compounds with good *in vitro* activity further to *in vivo* studies (Nuermberger, 2017). Relapse studies in mice, requiring sacrificing the animals are often performed later while *in vivo* PKPD with biomarkers can be run in parallel with *in vitro* studies (Margaryan et al., 2022). The complexity and costs of *in vivo* studies are the main reason to continue only with the compounds that show good activity in the *in vitro* analysis. The *in vivo* models range from the high-throughput, low-cost zebrafish, *via* the medium-throughput, medium cost mice, to the very low-throughput and expensive non-humane primates (Williams and Orme, 2016). Within this proposal for standardization of methodology, we have chosen only to address the murine models of the TB and the possible role they can play within the PK/PD evaluation. This is done, because the more rudimental *in vivo* models such as the (embryo) zebrafish can provide different steady state exposure results, but the limited duration of the experiments and the completely different PK, makes these models complementary to the mouse TB models (Ali et al., 2011) but not the ideal model for PKPD analysis, which is the focus of this overview.

First of all, it should be clear that mice and man are very different and that PKPD findings in mice should consider the transitional value in the preclinical drug-development. Within murine models of TB, we can study the PKPD relationship and assess the dose- or time dependent nature of the PKPD relationship trough dose fractionation studies. Essential in these studies is that besides dose, the actual drug concentration, preferably at the site of infection, is considered. Measuring the concentration of the (parent drug) compound and the (active) metabolites, *via* a chromatography based bioanalytical methods, the contribution of PKPD parameters can be made.

Preclinical murine TB models come in many different forms. The route of infection (e.g., intravenous, inhalation, or instillation) (de Steenwinkel et al., 2011), the inoculum size (de Steenwinkel et al., 2011), the mycobacterial strain used (Mourik et al., 2017), the pathology of TB in the specific model (Nuermberger, 2017), the treatment-free period before starting therapy and the mouse strain used, are all features that can be changed and tweaked to provide different models. There is no such thing as ‘the best murine TB model’, all models come with their own advantages and disadvantages. Thus, it seems reasonable to combine a set of preclinical models to inform the design of clinical trials. Broadly speaking there are acute, chronic and latent murine TB models to assess the effect of a treatment regimen. Acute models are informative on EBA and PKPD relationship, whereas the more chronic models assess the efficacy of therapy (prevention of relapse). Models for latent TB fall outside the scope of this paper (Zhang et al., 2011).

Regarding the PD (effect side) of the PKPD relationship, there are different outcome parameters that can be used. Traditionally, the number of colony forming units (CFU) are counted to the determine the mycobacterial load at specific that time-points and used as a biomarker to assess the PD in different organs. PD can also be assessed by time-to-positivity (TTP) measurement, using liquid cultures and automated hourly detection of growth or molecular load determination targeting rRNA, as read-out (de Knegt et al., 2017). Irrespectively of the method used to determine the PD or the type of animal model used, determination of the PK is essential to assess the PK/PD and thus the ability link the different phases of drug-development.

In order to conduct an adequate PKPD analysis using the *in vivo* experimental data, there should be gained insight in the exposure of the parent compound and the different metabolites and subsequently the contribution to the effect of these metabolites should be assessed. Although activity of metabolites has been tested *in vitro* experiments, the *in vivo* studies add additional information as in these studies activity of metabolites is included by nature. Given the different mode of action of the different compounds, the different metabolic states within which the different Mycobacteria are and the different immunological reactions to this infection, the diversity and complexity of these *in vivo* models are one step closer to that of TB patients. Bundling of the information of the different *in vivo* models in a model-based analysis is essential to strengthen the translational value of the different *in vivo* models (Margaryan et al., 2022; Mudde et al., 2022). In addition, the different animal models contribute with different information with respect to bactericidal and sterilizing drug efficacy.

Ideally, the outcomes of PKPD *in vivo* TB studies are informative in the way that human efficacy can be predicted for different drugs and regimens using model-based approaches for PKPD where information from human PK studies is linked to pre-clinical information about PKPD relationships and drug combinations (Wicha et al., 2018; Susanto et al., 2020; Mudde et al., 2022). This phase of the pipeline forms an essential biological bottleneck to increase the success rate of compounds or regimens to be entering the clinical development.

## 5 In human pharmacokinetics/pharmacodynamics: Early bactericidal activity

Early bactericidal activity (EBA) studies have been conducted since the landmark publication of Jindani et al. (1980), who tested a multitude of then available single agents and combinations over the first 14 days of treatment in small numbers of patients (Jindani et al., 1980). This study established EBA, defined as the fall in colony forming units (CFU) per ml of sputum over time, as a new method to quantify early drug effects. It also provided new insights into the clinical use of agents. Isoniazid (INH), for instance, was clearly the strongest drug in the first 2 days of treatment. Any treatment containing INH reduced the bacterial burden by 95% in 2 days, thereby lowering the risk of transmission (Mitchison, 2000). Its strong 2-day EBA is believed to make INH a good protector of companion drugs from acquisition of resistance because it can quickly eradicate any fast-growing mutants escaping control by other agents in a regimen (Mitchison, 2000).

It is generally accepted that patients with active pulmonary TB harbor a spectrum of bacterial phenotypes ranging from metabolically very active, fast growing, extracellular bacteria expectorated in sputum to more dormant, intracellular bacteria situated within granulomatous lesions. This requires different antibiotics with complementary characteristics to be part of a successful treatment regimen that can kill all these phenotypes (Dartois and Rubin, 2022). In the absence of the preclinical tools that we have available today, it was speculated at the time that strong 2-day activity would predestine a drug for killing mainly active, extracellular bacteria in the early stages of treatment, while other agents with more notable late EBA, such as rifampicin and pyrazinamide, would be sterilizing and contribute to curing patients (Mitchison, 2000).

The role of 14-day EBA studies in the clinical evaluation of novel antibiotics today is to determine whether a drug is active in humans, and to establish relationships between dose, drug exposure and bactericidal activity in small groups of patients (Diacon and Donald, 2014). EBA studies also give an early indication of safety and tolerability of new drugs and allow an indication of the combined effect of drugs given together. Fourteen days of monotherapy has been shown not to cause clinically relevant resistance (Kayigire et al., 2017). Extended EBA studies with strong combinations are limited by the reduced availability of viable bacteria for drug effect quantification. Even though all agents of our current first-line standard treatment regimens have significant 14-day activity it is not clear if and how EBA studies can predict the efficacy of combination regimens, at least not with the current standard biomarkers CFU and TTP.

Quantification of viable bacteria by CFU counting is hazardous, laborious, costly and relatively slow. Besides the recent introduction of TTP in liquid media, which is easier to standardize and more sensitive than CFU (Diacon et al., 2012b), a range of novel, not culture-based biomarkers are currently under investigation to better understand early drug effects (Heyckendorf et al., 2022). Analysis of EBA trials has historically been very diverse and not standardized, which has made it difficult to compare study results. Today, linking dosage, PK and PD data from multiple biomarkers creates an abundance of data and requires innovative analysis techniques.

Accordingly, a novel, model-based analysis methodology for EBA trials has recently been developed. Traditionally EBA was assessed as the observed difference in log_10_ CFU per ml of sputum between two study days (Jindani et al., 1980). Most commonly reported are 0-2-, 2-14- and 0–14-day intervals (Chan et al., 1992; Jindani et al., 1980; Sirgel et al., 1993). With liquid cultures, EBA is expressed as an increase in TTP over the same intervals (Diacon et al., 2012a). TTP is nowadays the preferred primary variable for EBA, with CFU as the secondary variable. Although CFU and TTP are highly correlated (Diacon et al., 2012b), EBA assessed with TTP can be followed for longer than with CFU. This is due to CFU only quantifying multiplying bacteria whereas TTP is thought to, in addition, capture the metabolism of non-growing bacteria and having a lower limit of detection.

Human studies are costly and thus missing information on an individual level through contamination or drop-out of patients can critically reduce the available information for calculating EBA from groups as small as 12–15 patients. Non-linear mixed effects modeling, in contrast, uses data pooled from all subjects, enabling all observations to contribute to the mathematical model. Missing information can be optimally handled. An innovative, standardized and model-based analysis methodology for analyzing EBA trials, accounting for known co-variates, has recently been developed and was used in the analysis of the EBA trial by De Jager (de Jager et al., 2022). From the model, predicted EBA intervals can be predicted, both on an individual level and population level but this is seen as a *post hoc* step in the approach. Instead, treatment differences for an EBA design with different regimens are evaluated on the model parameter TTP slope which is the parameter that describes the decline in TTP over time. The TTP slope can take on any function where a mono-exponential decline is the simplest. In order to assess the difference between two regimens, the model with no difference in TTP slope is compared for statistical difference to a model where there is a difference in slope between two regimen arms. This can be done for two arms or several arms. In addition to a point estimate in TTP, an imprecision in the EBA difference is obtained. The TTP model approach is therefore also suitable for assessment of efficacy in longer studies, for example seamless and adaptive Phase 2B/C studies where an early and informed decision is needed for potentially dropping arms in a study.

The standardized EBA modeling approach is also suitable for a PKPD approach where a PK summary variable such as AUC or C_max_ is used as a covariate. If there is high between-patient variability in PK, patients might be exposed to the same drug concentration even if they were given different doses or they may be exposed to different drug concentrations while receiving the same dose. Neglecting to collect PK data for such a drug in an EBA study may result in no difference in EBA if only the dose instead of drug exposure is used to evaluate differences in EBA. (Diacon et al., 2011).

Optimization of dosage for a single drug has been done for rifampicin where different monotherapy doses were studied over a 14-day period in order to determine the PK and assess EBA (Boeree et al., 2017; Svensson et al., 2018a; Svensson et al., 2018b). The dose optimization was mainly driven by the lack of PKPD evaluation of rifampicin during the clinical drug development. (van Ingen et al., 2011) However, EBA studies with regimens in addition to monotherapy arms, rather than only monotherapies are warranted in order to speed up clinical trial development as they are able to demonstrate synergistic effect between drugs which would otherwise go unnoticed (Diacon et al., 2012a). As such, the dose optimization and clinical trial prediction using preclinical information should rely on model-informed drug discovery and development (MID3) methods (Marshall et al., 2016).

## 6 Clinical pharmacokinetics/pharmacodynamics and therapeutic drug monitoring

In addition to the EBA studies there are several studies where PK/PD is used to answer specific questions related to drug exposure and treatment response. Clinical observations often provide the rationale for these studies and relate to treatment problems like slow response, relapse and acquired resistance. As in the clinical setting the source of these problems can be multifactorial, thus the design of a study needs to accommodate for all the potential sources that may contribute to the problem. When evaluating response to treatment low drug concentrations, drug resistance, non-adherence, and poor clinical condition and co-morbidities of the patient at the start of treatment all can explain the slow response (Alffenaar et al., 2022). Confounding factors can either be listed as exclusion criteria or can be appropriately quantified and documented to be included in the analysis of the study. Studies can be of observational nature explaining a specific outcome based on PK/PD or can be interventional of nature. In the latter one, dose optimization measures like dose escalation are studied and linked to the treatment outcome. In preparing both types of studies it is important to make use of available PK/PD data in conjunction with relevant clinical and microbiological information to guide a sample size calculation (Martson et al., 2020).

When and how PK should be assessed is guided by the hypothesis of the study or clinical question in routine care. For example, early assessment of PK is important when time to sputum culture conversion is assessed but also when studying acquired resistance. When the bacterial load is high, and bacteria are actively replicating there is a higher chance of development of resistance. In routine care, therapeutic drug monitoring (TDM) is used to assess the drug exposure and adjust the dose in individual patients.

TDM is a strategy that allows for dose individualization based on the measurement of drug concentrations in order to improve the efficacy of the treatment. The procedure includes identification of a patient that potentially benefits from TDM, collection of the appropriate timed samples to estimate the drug exposure, sample analysis, reporting and interpretation of the result, subsequent action which can be dose increase, decrease or decision to maintain the dose the same. Appropriate follow-up TDM needs to be scheduled to evaluate if the dose change has resulted in achieving the predefined target concentration. As the intention of TDM is to intervene where needed, the TDM cycle needs to be completed in a time manner. In TDM, the PK/PD index is compared to an established target where the dose can be increased if the PK/PD index is lower than the target or increased if the PK/PD index is higher than the target. (Alffenaar et al., 2022) From a traditional TDM point of view a sample collected 2 h and 6 h after drug intake are the relevant measures of drug exposure. (Alsultan and Peloquin, 2014) Using a sample collected 2rh after drug intake as surrogate for C_max_ is not sufficient as several studies have demonstrated that the time the C_max_ is achieved has a range of several hours (Sturkenboom et al., 2016). For example, the observed median T_max_ for rifampicin was 2.2 h, ranging from 0.4 h to 5.7 h, underpinning that multiple samples are required to capture the C_max_ (Sturkenboom et al., 2015).

Model-informed precision dosing (MIPD) is an approach where information from a population pharmacokinetic (POPPK) or physiology-based pharmacokinetic (PBPK) model in combination with individually observed plasma drug concentrations is utilized to forecast the dose that leads to the most optimal exposure in an individual patient (Darwich et al., 2017; Wicha et al., 2021) Further, MIPD can incorporate not only PK but also efficacy and safety aspects in the individual dose prediction, i.e. predict the dose given not only a POPPK or PBPK model, but also given PKPD models. When MIPD is used to perform TDM the drug exposure measures like AUC, C_max_ and C_min_ are relevant to calculate AUC/MIC, C_max_/MIC and %T > MIC. To be able to calculate these appropriate sampling schedules have to be designed. For the assessment of the AUC multiple samples are required. To adequately calculate an AUC using non-compartmental analysis 6-8 samples are required during the dosing interval (Gillespie, 1991; Gabrielsson and Weiner, 2012). Using a MIPD approach, flexible and different sampling schemes can be used and different exposure indicines (AUC, C_max_, drug concentration). When using population PK models in combination with a limited sampling strategy, 1-3 samples result in adequate accuracy of the AUC calculation (Sturkenboom et al., 2021). It is especially useful for drugs that exhibits large variability in exposure between occasions where it correctly can handle the between-occasion variability in exposure (Svensson et al., 2019a; Keutzer and Simonsson, 2020a). The MIPD approach can also be incorporated into mobile health solutions (Keutzer and Simonsson, 2020b; Keutzer et al., 2020).

The opportunities in routine care to assess the PD are often limited as bacteriological read outs are collected less frequently as in EBA studies. PD include sputum smear and sputum culture. In high incidence setting sputum is performed at start of treatment, after 2 months and close to the end of treatment. If sputum culture is performed, it is often limited to start of treatment and occasionally during treatment when suboptimal treatment is suspected. This limits the opportunity to assess the effect of differences in drug exposure on treatment response. The most frequently used PD endpoints are sputum culture conversion after 2 months of treatment (drug susceptible TB), and 6 months of treatment (drug resistant TB). Time to sputum conversion is relevant as it presents the same data more continuously than the fixed time points. When using the TTP, the bacterial load can be accounted for as well. The end of treatment is used to quantify treatment outcome (cure, failure, relapse, death, lost to follow-up). Subsequently the PK/PD parameter, taking into account drug exposure and pathogen susceptibility, are then correlated with the outcome measures. The simplest analysis includes the analysis of differences in treatment response between the different quartiles of drug exposures. Due to the limited sample size a statistically significant difference between the first and fourth quartile is often considered a proof of principle. More sophisticated regression methods like classification and regression tree analysis (CART) allow for the determination of thresholds associated with better (or worse) treatment outcomes (Zheng et al., 2021, 2022). It is important to account for differences in patient characteristics as earlier work identified a hard-to-treat patient phenotype which showed substantial lower cure rate and which may require longer treatment time compared to the easy to treat patients (Imperial et al., 2018).

Implementing TDM or MIPD poses significant challenges for programmatic care as measurements of drug concentrations need to be made available in a timely manner (Alffenaar et al., 2020). Multi-analyte assays are likely to be the most cost-effective approach when LC-MS/MS is used (Veringa et al., 2016; Kuhlin et al., 2019). However, as LC-MS/MS is not readily available in most high burdened settings alternative assessments can be of value. Dried blood spots are typically more stable and easier to transport to a central laboratory (Vu et al., 2011), but don’t solve the long turn-around time. More recently point-of-care tests using saliva (Alffenaar et al., 2021; Kim et al., 2021) or urine (Szipszky et al., 2021) have been developed to measure the drug exposure. When using targets from HFS-TB it is important that protein binding as well as drug susceptibility testing procedures are taking into account as MIC results may differ based on medium used. (Alffenaar et al., 2022) Likewise, when using saliva or urine are used for TDM targets in these alternative matrices need to be established or they need to reflect the corresponding plasma/serum target using a correction factor. For example, if the concentration in saliva is 80% of the plasma concentration the target concentration is 80% of the plasma target concentration or the saliva concentration needs to be multiplied by 1.25 to reflect the corresponding plasma concentration.

## 7 Modeling and simulation

Modelling and simulations are frequently used in the preclinical and clinical development, evaluation and optimization of therapeutic regimens and dosing schedules (Marshall et al., 2016; Wilkins et al., 2022).

Firstly, modelling approaches are used to characterize the PK of a TB drug in a population of interest. Thereby, pharmacometric approaches are increasingly used compared to conventional methods (two-stage approach, non-compartmental analysis) as they come with several advantages: pharmacometric models can handle sparse and/or imbalanced data. Thereby even PK data generated in routine TB care can be leveraged (van der Laan et al., 2021; Tietjen et al., 2022). Moreover, pharmacometric models allow to separate PK variability into different sources (e.g., within-patient, between patient, between dosing occasion, *etc.,*). Separation of PK variability, and in particular knowledge about potentialinter-occasion variability is key when the models should be used to calculate personalized doses using model-informed precision dosing, e.g., as shown for rifampicin by Keutzer et al. (Keutzer and Simonsson, 2020a). Likewise, patient covariates can be tested if they explain (parts of) the observed variability in PK. Pharmacometric models including patient covariates can be exploited for simulations. For TB, such approaches have specifically facilitated the evaluation of TB dosing regimens in children, where the impact of body weight and organ maturation is included (Zvada et al., 2014). Also, pharmacometric models have an important role in the evaluation of drug interactions, e.g., with anti-retroviral drugs (van der Laan et al., 2021) in the co-treatment of HIV and TB.

Secondly, modelling approaches are used to characterize the exposure-response relationship of TB drugs (Wilkins et al., 2022). For this purpose, traditionally CFU or TTP are utilised, but biomarkers such as the molecular bacterial load (MBL) are also considered (Honeyborne et al., 2011). Modelling approaches have proven useful to define the links between different biomarkers, e.g. for MBL and TTP in liquid culture (Svensson et al., 2019b). Exposure-response analyses are challenging in TB since TB drugs are only used as monotherapy in dose-ranging EBA studies for a short duration up to 2 weeks (Te Brake et al., 2021). Phase 2b/c trials typically place novel TB drugs into a combination treatment, which makes exposure-response analyses challenging. Moreover, the relationship between relapse and the biomarkers that are detectable only in the early phase of treatment is not conclusively understood. Non-etheless, for example for bedaquiline, using an integrative pharmacometric model that characterized the population PK as well as comprised a time-to-event model to characterize the TTP, Svensson et al. successfully described bedaquilines’ exposure-response relationship in data from a phase 2b trial (Svensson and Karlsson, 2017). Modelling of previously acquired clinical data could also help to streamline the design of future phase 2b studies. For example, Gewitz et al. (2021) analysed data from two phase 2b studies on rifapentine. The authors did not only describe the exposure-response of rifapentine in these data, but also performed a sensitivity analysis, which revealed that significant exposure-response and covariate relationships could be detected using truncated TTP data as early as 6 weeks from start of treatment, suggesting alternative phase 2b designs.

In case of safety concerns, pharmacometric models can quantify relationships between a variable that quantifies the side effect and PK. For example, Tanneau et al. (2021) investigated the link between the QTc interval, transaminase levels and bedaquiline exposure in phase 2b clinical data. The concentrations of the bedaquiline M2 concentrations were found to be responsible for the drug-related QTc increase, whereas no exposure-safety relationship was found with transaminase levels despite previous reports of higher levels in patients treated with bedaquiline. In another very illustrative example, Imperial et al. (2022) developed a pharmacometric model based on the Nix-TB trial (NCT02333799) including the toxicodynamics of linezolid. The authors identified links between linezolid exposure and the frequency of thrombocytopenia, neuropathy and anaemia and defined practical markers to guide dose adjustments in the treatment course.

Thirdly, modelling approaches are by no means limited to clinical data and are increasingly used to quantitatively characterize data stemming from pre-clinical TB studies. Indeed, in particular *in vitro* studies using time-kill curve studies, or the hollow-fiber infection model allow for a detailed characterisation of the PK/PD of TB drugs alone and in combination. For example, Clewe et al. (2016) developed the multi-state TB pharmacometric (MTP) model to quantify the effect of rifampicin on fast-, slow- or non-growing mycobacteria. Coupled to PK data and accounting for system-specific variables, the MTP could be used to predict across different preclinical systems up to EBA studies for rifampicin (Wicha et al., 2018), and/or isoniazid (Susanto et al., 2020) in addition to describing semi-mechanistic PKPD relationships in humans (Svensson and Simonsson, 2016; Faraj et al., 2020). Also, the study by Kim et al. (2022) defined the effect of clofazimine in combination with pretomanid, bedaquiline or linezolid against various growth states of TB and profiled their potential for synergistic interactions. Modelling approaches can also provide quantitative insights into data originating from mouse models (Chen et al., 2017; Wagh et al., 2021). For example, for spectinamide 1810, modelling helped to characterize the PK/PD indices of this new drug candidate and a model accounting for the post-antibiotic effect was developed, which might help to select adequate dosing regimens for future efficacy studies (Wagh et al., 2021). In addition, correlation between treatment length and treatment outcome is an important aspect of *in vivo* studies (Mudde et al., 2022), which is required to efficiently estimate a regimens’ treatment-shortening potential. Mourik et al. (2018) developed a new design for *in vivo* relapse studies together with a pharmacometric approach for prediction of probability of cure given different treatment lengths of regimens (Pieterman et al., 2021; Mudde et al., 2022).

In summary, modelling approaches to characterize PK are already highly standardized whereas the model-based characterization of the PK/PD needs to be tailored to the research question and system of interest. Non-etheless, modelling and simulations provides the opportunity to integrate data along the drug development pathway (Bartelink et al., 2017; Wicha et al., 2018; Radtke et al., 2021), which may help to better understand the PK/PD from a quantitative perspective and bridge the translation from pre-clinical to clinical research.

## 8 Conclusion

In this review we provided an overview of pre-clinical and clinical methods to assess the PKPD of anti-TB drugs (Table 2). By in-depth discussion of design of *in vitro*, *in vivo*, and human studies and subsequent strategies to appropriately integrate the data from these studies using mathematical models, we contribute to debate how to accelerate the translation of findings to clinical practice*. In vitro* static time-kill experiment and dynamic models are to be used for early identification of PKPD relationships and favorable drug regimens. Similarly, simple *in vivo* models (zebra fish) can be used for drug screening studies while rodent models can help to understand PKPD relationships of the drugs in relation to treatment outcome and relapse. Results from adequate *in vitro* and *in vivo* studies can help to guide dose selection for 14-day EBA studies. These studies will determine drug activity and PKPD relationships and safety in humans. Further confirmation of PKPD in large scale phase 3 studies will help to create a better understanding of variability in drug exposure and implications for treatment outcome and hence can contribute to further dose optimization in subpopulations or personalized dosing *via* TDM or MIPD. Modelling and simulations provide the opportunity to integrate data from *in vitro*, *in vivo* and clinical studies, which contributes to a better understanding of the PKPD of a drug and has the opportunity to accelerate the clinical development pathway.

**TABLE 2:** Key aspects of PKPD studies.

Study	Study design	TB	Participants	PK	PD	Application
*In vitro*	Intracellular	H37Ra H37Rv Clinical isolates	cells	Sampling of system (multiple)	CFU	*Define PKPD parameters for optimal kill, prevention of resistance
	Extracellular					
*In vivo*	Balb/c		animals	1 sample/animal, different time points different animals	CFU	*Define PKPD parameters for cure, prevention of resistance, relapse
	C3Heb/Fej					
EBA	Selected TB patients with drug susceptible TB	Clinical isolates	patients	Full PK curve	CFU, TTP	Confirm activity in humans, confirm PKPD relationship, define dose for phase 2b
					Daily for 14 days	
Phase 2/3	Selected TB patients with drug susceptible or drug resistant TB	Clinical isolates	patients	Limited samples in all participants, Full PK curve in subset of the patients	Culture conversion, treatment outcome	Define dose for regulatory approval
TDM	Clinical practice	Clinical isolates	patients	Ranging van limited samples to full PK curve	Successful treatment of individual patients	Define subpopulations, situations where standard dose is likely not effective or toxic

TB, tuberculosis; PK, pharmacokinetics; PD, pharmacodynamics; CFU, colony forming units; TTP, time to positivity; TDM, therapeutic drug monitoring; *The MIC of the strain used in the *in vitro/in vivo* study should be determined before each experiment as well as the MIC of the clinical strains to determine if the given clinical dose could achieve the PK/PD exposure target in patients.
